# Dr Christina Yen contemplates and imparts the bioethical dimensions of stewardship and infection prevention

**DOI:** 10.1017/ash.2023.414

**Published:** 2023-08-22

**Authors:** Christina Yen

**Affiliations:** Division of Infectious Diseases and Geographic Medicine, Department of Internal Medicine, University of Texas Southwestern Medical Center, Dallas, TX, USA

1. Tell us about your unique background and training

I was an anthropology major with a bioethics focus and an occupational science minor at the University of Southern California (USC). From this interdisciplinary background, I viewed medicine as a culture with its own practices, values, and beliefs. I stayed on at USC for medical school and internal medicine residency. My role models in residency at the Los Angeles County hospital inspired me to pursue healthcare epidemiology and antimicrobial stewardship and encouraged to go somewhere with dedicated fellowship training in these areas. I was fortunate to match at Beth Israel Deaconess Medical Center’s (BIDMC) infectious diseases fellowship and stayed for an additional year of training in hospital epidemiology, infection prevention, and antimicrobial stewardship. I completed the Program for Clinical Effectiveness through the Harvard TH Chan School of Public Health which included biostatistics and epidemiology courses and am happy to report that I just completed my Masters in Bioethics through the Harvard Medical School in May 2023!

1. How did early relationships and exposure to ID pharmacists help shape your career path?

As a resident, I watched ID attendings regularly involve pharmacists and trainees on rounds. This shaped my understanding of ID pharmacists as partners in providing safe, high-quality patient care and as highly educated experts with complementary skills. As a result, I loved spending time with ID pharmacists, and I saw this as an opportunity to receive extra teaching. You can imagine then that my co-residents were all too happy to let me take the lead on calling for antibiotic approvals.

My appreciation for ID pharmacists as teachers, role models, and partners in patient care expanded during fellowship. This was especially true as I learned more about the logistics of running an antimicrobial stewardship program. The experiences and discussions I had with ID pharmacists during my training inspired my bioethical examination of our discipline. I truly believe that my career has been enhanced by appreciating the complementary nature of our fields, developing communication skills that acknowledge cross-professional similarities and differences, and advocating for greater acknowledgment of their work and value.

1. What life and professional experiences led you to study the bioethical dimensions of stewardship? Were you always interested in bioethics and medicine? How did you find this path?

Bioethics and the social science aspects of medicine have always attracted me, but I reacquainted with them as my primary academic focus during the COVID-19 pandemic.

I joke that I was self-stewarding at an early age, like the hopeful ID candidate in the Dr Glaucomflecken satire. In my youth, I was regularly prescribed antibiotics for my asthma exacerbations and respiratory viral infections but would avoid taking them like many children who dislike medications.

As an undergraduate, I enjoyed my anthropology, sociology, and ethics courses the most, therefore assumed I would either become a medical anthropologist or occupational therapist. I didn’t see many medical school applicants with humanities or social science backgrounds, so I was doubtful of my potential. I later discovered those studies enriched my medical training. Bioethics was everywhere: daily interactions with patients, dialogs between physicians and other healthcare professionals, or end-of-life discussions. This awareness provided a unique view of medicine and gave me the tools to support families in making tough decisions during the anticipated or unexcepted deaths of my own family members.

The COVID-19 pandemic ignited my pursuit of an academic career in bioethics. At the onset, I was completing an additional year of training in infection control, healthcare epidemiology, and antimicrobial stewardship. I observed that decision-making was greatly informed by ethical considerations since data was limited or rapidly emerging. At baseline, infection control, epidemiology, and stewardship had limited incorporation of bioethics. Bioethicists were engaged to make decisions about masking, quarantine, or antiviral allocation, but often unaware that expertise like ours existed or could be harnessed for help. I saw this gap as an opportunity to utilize my skills and passions to contribute a new perspective.

1. What is bioethical epistemology and what should all stewards know about it? How should we be thinking about these topics to unlock the maximal potential of stewardship?

Epistemology is defined as the theory of knowledge; in short, how do we know what we know? Questions that epistemological inquiry seek to answer include: How do we determine what is true or right? How do we obtain that knowledge? How do we vet our sources?

One way to unlock the potential synergy of bioethics and stewardship is to deeply interrogate fundamental beliefs. My favorite example is the question “what do we mean when we say, ‘judicious’ antimicrobial usage?” We are quick to recognize poor prescribing, but “appropriate” prescribing is harder to describe.

Because judicious antimicrobial use is the primary goal of antimicrobial stewardship, all stewards have an opinion of what that means. I’ve asked this question to many people, and I never tire of the varied answers I receive. Some focus more on the need to slow antimicrobial resistance, others on changing prescribing behavior. For a special few, antimicrobial usage is interwoven into healthcare, or entire social systems. There are others who return to the root word, “justice,” ie, for whom is this usage fair? Who gets to decide what judicious prescribing looks like? These interpretations of “judicious” urge us to consider issues of equity, representation, and bias as it relates to antimicrobial prescribing and resistance. In fact, I would invite the readers of ASHE to consider this question as it relates to their work. I think it will surprise many of us to discover the latent ethical dimensions of our work!

1. Describe a bioethical challenge that stewards, infection preventionists, and epidemiologists commonly face and how can we apply a bioethical framework to finding solutions?

This challenge is eloquently described by “The Patient as Victim and Vector” by Margaret Battin *et al*. It is a collection of essays primarily by ethicists and health policy experts on the ethical dilemmas as pertains to infectious diseases. This duality of victim and vector speaks to an infected patient being simultaneously a risk to others and at risk of morbidity and mortality. How do we balance these factors? How do we implement interventions that walk that balance?

For epidemiologists and infection preventionists, isolation precautions and quarantine are prime examples as we witnessed during COVID-19 and mpox. This dilemma arises during the initiation and de-implementation of intervention. Prior authorization of empiric antimicrobials in stewardship is another example. These decisions require quantitative data as well as considerations for a patient’s autonomy, public welfare, awareness of historic and existing oppression, our own biases, and many more ethical dimensions that are intuitively incorporated in the calculus of developing guidelines but are often neither explicitly named nor examined when making hard decisions in these fields.

1. What is the connection between antimicrobial resistance and bioethics? Can this be expanded further to other important areas such as health equity?

This question alone is the basis for a future commentary. Those who have written extensively on the connection between antimicrobial resistance and bioethics, like Otto Cars, Jasper Littmann, and Euzebiusz Jamrozik, are bioethicists, health policy experts, etc, but none are antimicrobial stewards or ID clinicians. Therefore, I seek to build upon their work and will attempt to distill some points for ASHE readers:

The prevalence of multidrug resistant infections and their consequences increase every year, with a projected 10 million human deaths a year by 2050, which is equally impactful to animals and the environment. Given the extent of the issue, we can safely call it a public health crisis. Fiscally, AMR is a disaster: CDC estimating that AMR costs 20 billion dollars in direct health costs and 35 billion from productivity loss. Furthermore, the brunt of AMR’s burden is shouldered by the most vulnerable: the clinically vulnerable, the socioeconomically disadvantaged such as those living in LMICs, the racially oppressed and stigmatized. This renders AMR a clinical, societal, and geopolitical crisis, too. Those who need measures to slow AMR, such as clean water and sanitation, access to newer antimicrobials, are often the least able to access them. This renders AMR a challenge to health equity and healthcare access.

Because AMR and antimicrobials are deeply interwoven into the function and dysfunction of modern society, curbing the acceleration of AMR goes beyond a stern phone call to a physician who wants empiric vancomycin. AMR challenges us to consider hard ethical questions about justice, autonomy, resource allocation, our responsibilities to future generations; to name a few!

1. As someone who is comprehensively trained in infection prevention, healthcare epidemiology and stewardship, how important is it to maintain skills in all areas versus differentiating further in one specific area to advance one’s career?

While it has been immensely rewarding to develop new expertise, I find myself routinely returning to my foundational skills. First, because I am still in the early stages of my career; I still have plenty to learn and refine. Second, I have always wanted my work to be useful to everyday ID clinicians and stewards; therefore, it is necessary to maintain these skills for my work to serve those who need it most. From these daily interactions with patients, clinicians, and stewards, I draw inspiration for bioethical inquiries.

1. You have a keen focus on trainees and medical education—how can we do a better job of incorporating bioethics and social sciences in residency or fellowship training?

By acknowledging the need for greater integration of bioethics and social sciences into medical training, we’re already making progress. One way we can improve is to engage our residents and fellows in their own bioethical education. More trainees are entering medicine with a background in the social sciences or humanities. Not engaging them is a missed opportunity to move the field forward.

You’re still early in your career but have taken on an active role in mentoring others. Tell us about pivotal mentorship experiences that have shaped your career (feel free to mention specific names) and how these experiences defined your approach to mentoring

I will limit myself to three experiences with different mentors which greatly impacted my career, although I can think of many.

One of my earliest experiences was with Dr Brad Spellberg an internal medicine resident at Los Angeles County Hospital. Doing research with Brad helped me develop good research skills and habits. His willingness to involve me in stewardship projects enhanced my enthusiasm for ID and changed the course of my career in antimicrobial stewardship and healthcare epidemiology. This experience taught me the value of early mentorship, so I try to engage interested residents and fellows early in their training!

Drs Monica Mahoney and Nick Mercuro are two ID Pharmacy leaders who took me under their wing. I shared with Monica my interest in writing and editing; she took me through my first manuscript reviews and has regularly sponsored me ever since. Nick Mercuro is a stellar research mentor, his ability to develop thoughtful, practical research questions helped me refine my earliest interdisciplinary inquiries.

Lastly, my experience with mentors like Brad Cutrell, Preeti Mehrotra, and Payal Patel has taught me the value of exploring new areas of knowledge as a mentor/mentee team. Preeti and Payal do work in interdisciplinary spaces to expand the knowledge base in healthcare epidemiology, infection prevention, and antimicrobial stewardship. Although bioethics is not Brad’s area of focus, he was always willing to learn along with me as one of my master’s capstone project mentors. I now have mentees with interdisciplinary interests like critical care/antimicrobial stewardship, and I find that their passions spark my own academic curiosity.

1. Finally, as someone who works so closely with pharmacy colleagues, how can ID docs, infection preventionists, and other healthcare professions champion the work of our ID pharmacy colleagues, uplift them, and help them feel seen and heard?

Recently I’ve spent time thinking about how I may inadvertently perpetuate harm. None of us like thinking of ourselves as causing harm given that our field is rooted in the Hippocratic oath. However, until we can examine our own shortcomings, it is hard to claim true interprofessional investment in our ID pharmacist colleagues.

For example, we’ve all observed that some may be resistant to the recommendations of our ID pharmacist colleagues until ID physicians intervene. While my intervention ensured the desired result, I did not take the opportunity to ask why the original recommendations were ignored. Was it because the rationale did not resonate with the physician? Was it because of the ID pharmacist’s terminal degree? Their nonclinician status? I realize now that my silence was a form of permission for other clinicians to continue this behavior and, in turn, caused inadvertent harm to my ID pharmacist colleagues. Physicians continue to possess significant power in the hierarchy of medicine; we can use it to inquire and correct assumptions about our ID pharmacy colleagues and shift the power dynamics. Now, I take a moment during such stewardship interactions to emphasize that my recommendation is identical to that of my ID pharmacy partners.

